# Pediatric bacterial meningitis in southern China: analysis of 838 cases

**DOI:** 10.3389/fcimb.2025.1481716

**Published:** 2025-02-05

**Authors:** Lianfeng Chen, Wen-Lin Wu, Yuanyuan Gao, Xiaojing Li, Sida Yang, Huici Liang, Kelu Zheng, Yani Zhang, Haixia Zhu, Yang Tian, Bingwei Peng, Haisheng Lin, Xiuying Wang, Shuyao Ning, Yinyan Gan, Chi Hou, Yinting Liao, Huiling Sheng, Wen-Xiong Chen

**Affiliations:** ^1^ Department of Neurology, Guangzhou Women and Children’s Medical Center, Guangzhou Medical University, Guangzhou, China; ^2^ Department of Behavioral Development, Guangzhou Women and Children’s Medical Center, Guangzhou Medical University, Guangzhou, China

**Keywords:** bacterial meningitis, clinical features, children, prognosis, risk factors

## Abstract

**Objective:**

This work aims to study the clinical features and risk factors of children with bacterial meningitis (BM) in southern China.

**Methods:**

Clinical data of children with BM between 2012 and 2018 from one national center were analyzed retrospectively.

**Results:**

A total of 838 patients (male/female = 1.8:1) were enrolled, with 90.6% under 1 year old. Common symptoms included fever, seizure, lethargy, vomiting, anorexia, poor feeding, and irritability. Most patients initially exhibited typical cerebrospinal fluid (CSF) changes of BM, including elevated white blood cell count, increased protein levels, and decreased glucose concentration. Some initially atypical cases showed typical changes after about 1 week. Furthermore, 38.7% of the patients had positive bacterial cultures of blood or CSF, with *Streptococcus agalactiae*, *Escherichia coli*, and *Streptococcus pneumoniae* commonly seen. Moreover, 92.0% of the patients were graded five Glasgow outcome scale (GOS) points at discharge. Differences in symptoms, pathogens, CSF results, brain MRI, and GOS points were observed across age groups (neonate [29 days, 12 months) and aged ≥12 months). Fatality rate was 1.9%, and 10.7% of survivors had neurological sequelae. Recurrent BM was rare (1.6%) but notable in patients with CSF fistula or immunodeficiency. Risk factors for intensive care unit admission, brain parenchymal involvement, subdural effusion, and hearing impairment were identified.

**Conclusion:**

Most pediatric BM patients in southern China were under 1 year old, with more distribution in male patients and some age-related differences in clinical features and outcomes. Recurrent BM is rare but more likely in patients with conditions such as CSF fistula or immunodeficiency. Most patients have favorable outcomes, with a low fatality rate and around 10% of the survivors experiencing neurological sequelae. Several clinical risk factors were identified.

## 1 Introduction

Bacterial meningitis (BM) is a severe infectious disease, especially for neonates and children, resulting in high mortality and morbidity associated with diagnosis and treatment delay (Li et al., 2014; Lucas et al., 2016; van de Beek et al., 2016b; Zainel et al., 2021). Therefore, early diagnosis and appropriate antibiotic treatment are important for patients with BM. Although the incidence of BM caused by *Streptococcus pneumoniae*, *Neisseria meningitidis*, and *Haemophilus influenzae* has decreased significantly worldwide in the past two decades with the use of vaccines for these three pathogens, BM remains a common and acute, devastating infection in neonate and children (McIntyre et al., 2012). In addition, the sometimes inappropriate use of antibiotics leads to atypical changes in the cerebrospinal fluid (CSF) of partial BM patients, complicating timely diagnosis (Subspecialty Group of Neurology tSoPCMA, 2019). In this study, we reported the clinical features of 838 children diagnosed with BM, and we also explored the risk factors of admission to the intensive care unit (ICU), brain parenchymal involvement, subdural effusion, and hearing impairment in a national, regional tertiary medical center from southern China in the past 6 years (from 2012 to 2018).

## 2 Subjects and methods

### 2.1 Subjects

Children diagnosed with BM from October 2012 to September 2018 in the department of neurology of Guangzhou Women and Children’s Medical Center were included. The clinical features of patients, including demographic data, prodromal factors, clinical manifestations, laboratory investigations, neuroimaging examination, neuroelectrophysiological data [electroencephalogram (EEG) and audiological assessment], comorbidity, complications, treatment, outcomes, and prognosis were retrospectively reviewed. The patients were grouped according to their age at onset: neonate, [29 days, 12 months), and ≥12 months. The closing parenthesis (“)”) indicates “less than”, and the square bracket (“[“) indicates “greater than or equal to”. Clinical outcome was defined as clinical status on the day of discharge, and the outcome was graded with the Glasgow outcome scale (GOS) (1): death (1 point), (2) persistent vegetative state (2 points), (3) severe disability (3 points), (4) moderate disability (4 points); and (5) good recovery (5 points). A good outcome was defined as a GOS score of 5, and a poor outcome was a GOS score of 1 to 4 (Guo et al., 2016). This study was approved by the Ethics Committee of Guangzhou Women and Children’s Medical Center (approval no.: 2019052419364384). Written and signed consent was obtained from the patient’s parents or guardians, who also explicitly consented to publish their details, clinical data, and images that could identify them. A total of 70 patients with bacterial meningitis caused by *Streptococcus agalactiae*, *Streptococcus pneumoniae*, or *Escherichia coli* from 2015 to 2018 in the neurology department in one of our branch hospitals has been reported in a previous study (Chen et al., 2022).

### 2.2 Methods

#### 2.2.1 Inclusion criteria

Patients aged ≤14 years, including newborns, diagnosed with BM with proven bacteria, BM with negative bacterial culture, probable BM, or suspected BM were involved (per diagnosis criterion seen below).

#### 2.2.2 Exclusion criteria

The exclusion criteria were (1) encephalitis or meningitis caused by pathogens other than bacteria or (2) autoimmune neurological disorders.

#### 2.2.3 Diagnosis criteria

Diagnosis of BM: BM with proven bacteria was defined as positive bacteria in the CSF or blood culture with compatible symptoms and signs of meningitis (Guo et al., 2016). BM with negative bacterial culture was defined as (1) both CSF and blood bacterial cultures were negative and (2) symptoms and signs compatible with BM with at least three of the following four criteria: (a) CSF WBC >1,000 × 10⁶/L; (b) CSF glucose level <2.2 mmol/L; (c) CSF protein concentration >0.45 g/L); and (d) serum CRP >40 mg/L (Peltola et al., 2010). The diagnostic definitions of “probable BM” and “suspected BM” used in our study are based on the criteria set by the World Health Organization (WHO), as outlined in previous studies, including Zhu et al. (Guo et al., 2016). Probable BM was defined as (1) not meeting the criteria of BM with positive or negative bacterial culture and (2) symptoms and signs compatible with BM with at least one of the following three criteria: (a) cloudy CSF appearance; (b) CSF WBC >100 × 10⁶/L; (c) CSF WBC of 10 to 100 × 10⁶/L with either an elevated protein level (>0.45 g/L) or (d) CSF glucose level < 2.2 mmol/L (Guo et al., 2016). The suspected BM was defined as (1) sudden onset of fever (>38.5°C rectal or >38.0°C), (2) neck stiffness, altered consciousness, or other meningeal symptoms (Guo et al., 2016). Recurrent BM was defined as two or more episodes of meningitis caused by a different bacterial organism or a second or further episode caused by the same organism with a greater-than-3-week interval after the completion of therapy for the initial episode (Tebruegge and Curtis, 2008).

Diagnosis of immunodeficiency: Patients with BM in combination with immunodeficiency disease, primary immunodeficiency disease, and X-linked agammaglobulinemia were diagnosed according to the European Society for Immunodeficiencies criteria (Diagnostic criteria for PID, 2018).

#### 2.2.4 Classification of hearing impairment

Hearing impairment was classified according to the threshold of brainstem auditory evoked potential (BAEP) as mild [26–40 dB], moderate (40–60 dB], severe (60 dB–80 dB], or profound (>80 dB) according to the International Classification of Impairments, Activities, and Participation (Lucas et al., 2016).

Neonate hearing screening was evaluated by transient-evoked otoacoustic emissions (TEOAE) test performed using Bio-logic^®^AuDX^®^ (Natus Medical Incorporated, Middleton, WI, USA). TEOAE pass was considered as normal; otherwise, it was considered abnormal. TEOAE pass criteria included ≥70% whole wave reproducibility and signal-to-noise ratio ≥6 dB in the center frequencies from 1,236 to 3,536 Hz.

#### 2.2.5 Treatment

Empiric antibiotic treatment was given to all patients before the causative bacterial was identified, and the antimicrobial agent was adjusted according to the susceptibility results if the pathogen was identified. Dexamethasone (0.15 mg/kg day per 6 h for 4 days or 0.4 mg/kg day per 12 h for 2 days) was used among patients at an early acute stage. Neurosurgical intervention was given to patients with subdural effusion, subdural empyema, brain abscess, hydrocephalus, or structural abnormalities of the brain or spine when needed after consultation with neurosurgical doctors.

#### 2.2.6 Follow-up

All patients were followed up either by a neurologist in the neurological clinic or by a neurologist via telephone contact.

#### 2.2.7 Statistical analysis

Statistical analysis was performed using SPSS IBM 20.0. Quantitative data with normal distribution was described by mean ± SD; otherwise, it was median with the interquartile range (IQR). The qualitative data was described by frequency and percentage. Pearson chi-square, likelihood ratio, or Fisher’s exact test was used to compare the qualitative data. The quantitative data with normal distribution were compared using independent *t*-test or analysis of variance with *post hoc* by Student–Newman–Keuls *q* test; otherwise, Mann–Whitney *U* or Kruskal–Wallis *H* test with *post hoc* by Nemenyi test was used. Univariate logistic regression (LR) analysis was conducted to determine the ability of significant risk factors. Missing data were handled using multiple imputation techniques. A follow-up forward stepwise LR was then performed that included risk factors with a cutoff of *p <*0.1 in the univariate LR analysis. A *p*-value <0.05 (two-sided) was considered significant. Figures were graphed using GraphPad Prism 7.01 (GraphPad Software Inc., USA).

## 3 Results

### 3.1 Demographics

A total of 838 patients (male/female = 1.8:1) were enrolled, and all patients were Chinese except for two African patients. There were 572 patients who were initially diagnosed with BM in our hospital, and the other 266 patients were initially diagnosed with BM in other hospitals and then transferred to our hospital. The median onset age was 1 month (IQR 18 days–3 months, ranging from 1 day to 13 years). Most patients were under 1 year old, accounting for 90.6% (759/838), especially newborns and infants under 3 months, accounting for 72.9% (611/838). The age distribution of the patients was as follows: ≤28 days (40.0%, 335/838), [29 days, 3 months) (32.9%, 276/838), [3 months, 12 months) (17.7%, 148/838), [12 months, 3 years) (3.4%, 28/838), [3 years, 5 years) (2.5%, 21/838), and ≥5 years (17.7%, 30/838), respectively. The high-occurrence season was summer (37.0%, 310/838), followed by spring (22.4%, 188/838), autumn (20.1%, 168/838), and winter (20.5%, 172/838) respectively. Moreover, 64.2% (538/838) of patients were vaccinated on time, and 66.7% (559/838) of patients received antimicrobial therapy within 3 days before admission.

### 3.2 Clinical features

#### 3.2.1 Prodromal factors

Among the patients, 30.2% (253/838) had prodromal factors, with infectious prodromal factors being the most common (*n =* 176), followed by perinatal prodromal factors (*n =* 71) and trauma (*n =* 6). Respiratory infectious symptoms were the most common infectious prodromal factors seen in 116 patients (65.9%, 116/176), followed by infectious digestion symptoms (25.0%, 44/176), acute tympanitis (*n =* 8), urinary tract infection (*n =* 7), and renal abscess (*n =* 1). For patients aged <2 months, 13.8% (71/513) of them had perinatal prodromal factors before onset, with maternal premature rupture of membrane being most commonly seen (62.0%, 44/71), followed by moderate to severe turbid amniotic fluid (32.4%, 23/71), and maternal vaginal and perianal swabs that tested positive for *Streptococcus agalactiae* (5.6%, 4/71). Six patients had trauma before onset, including head trauma (*n =* 4, respectively occurring at 2 months, 3 months, 6 months, and 3 years before onset) and nasal trauma (*n =* 2, 2 to 3 days before onset).

#### 3.2.2 Clinical manifestation

Clinical symptoms and sign: The common symptoms included fever (92.0%), seizure (32.2%), lethargy (18.3%), vomiting (14.3%), anorexia and poor feeding (12.1%), and irritability (11.8%). The less common symptoms included tachypnea (8.6%), startled (6.1%), impaired consciousness (5.5%), groaning (5.4%), headache (3.8%), cyanosis (2.9%), apnea (2.1%), skin mottling and cold extremities (1.9%), and movement disorders (paralysis and/or dystonia, 1.8%). The common signs included bulging anterior fontanel (16.1%) and stiff neck (7.6%).

**Figure 1:**
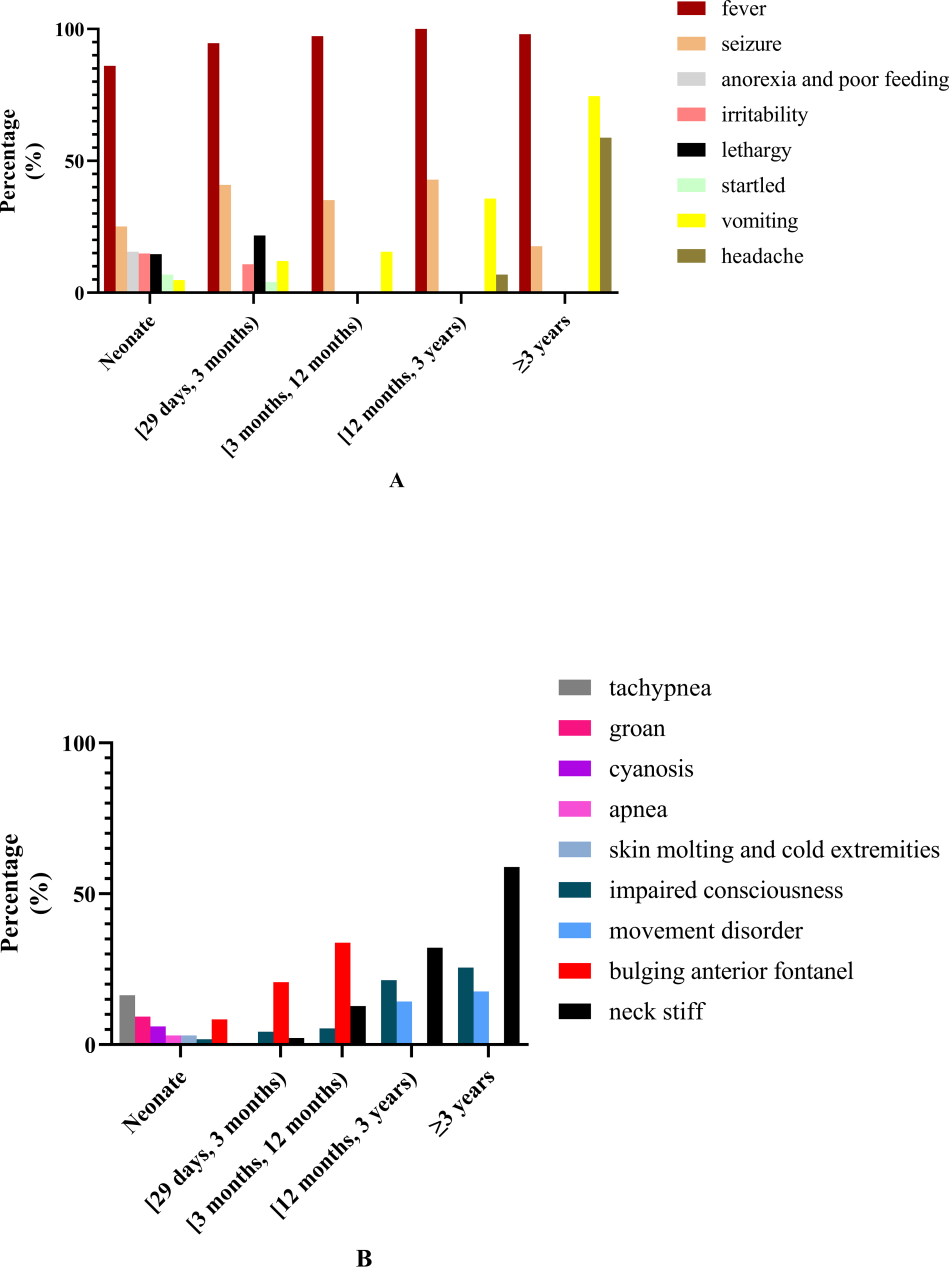
Common and less common symptoms and clinical signs in patients of different ages. **(A)** Common and less common symptoms in patients of different ages. **(B)** The common and less common clinical signs in patients of different ages.

Clinical manifestation in patients of different age groups: The common and less common symptoms in patients of different age groups are shown in **Figure 1**. Fever was the most common symptom in all patients of different age groups. The second most common symptom in patients aged <3 years was seizure, while in patients aged ≥3 years it was vomiting. The frequency of vomiting increased with age. Dyspnea was more commonly seen in neonate than non-neonate patients (26.1% vs. 7.0%, chi-square test, *χ* ^2^ = 59.873, *P* < 0.0001).

### 3.3 Ancillary test results

#### 3.3.1 Peripheral blood test results

All patients underwent routine blood and C-reactive protein (CRP) tests when first admitted to our hospital. The median blood white blood cell (WBC) count was 11.7 × 10^9^/L (IQR 8.3 × 10^9^/L–16.8 × 10^9^/L, ranging from 1.3 × 10^9^/L to 42.6 × 10^9^/L) (normal reference range: 4.0 × 10^9^/L–12.0 × 10^9^/L). Blood WBC higher than the upper limit of normal values (ULN) was most commonly seen in 49.5% (415/838) of patients, including blood WBC in the range of 12.0 × 10^9^/L to 20.0 × 10^9^/L (34.4%) and ≥20.0 × 10^9^/L (15.2%), followed by within normal reference range (45.0%, 377/838), and lower than the lower limit of normal values (LLN) (5.5%, 46/838). The median CRP was 36.49 mg/L (IQR 3.44–117.4 mg/L, ranging from 1.00 to 329.06 mg/L) (normal reference value: ≤8.2 mg/L). CRP higher than ULN was seen in 69.2% (580/838), with CRP >100 mg/L most commonly seen in 28.9%, followed by CRP belonging to (8.2 mg/L, 50.0 mg/L) (26.0%) and [50.0 mg/L, 100.0 mg/L] (14.3%). There were no significant differences in blood WBC and CRP in patients of different age groups (more details are shown in **Table 1**).

**Table 1:** Clinical features of children with bacterial meningitis.

	Total (*n =* 838)^#^	Neonate (*n =* 335) ^#^	[29 days, 12 months) (*n =* 424) ^#^	≥12 months (*n =* 79) ^#^	*P*
Male/female	1.8:1	1.7:1	1.8:1	1.8:1	0.606^a^
Blood WBC (median (IQR), ×10^9^/L)	11.7 (8.3, 16.8)	12.1 (8.6, 17.2)	11.2 (8.1, 15.8)	12.6 (8.4, 18.0)	0.066^b^
Blood CRP (median (IQR), mg/L)	36.5 (3.4, 117.4)	32.3 (3.4, 87.2)	37.6 (3.4, 128.9)	49.9 (3.92, 159.4)	0.153^b^
CSF WBC (median (IQR), ×10^6^/L)	150 (40, 650)	214 (50, 1,056)^#^	101 (30, 384)^*^^	390 (110, 1,186)^#^	<0.001^b,c^
CSF glucose (mean ± SD, mmol/L)	1.76 ± 1.11	1.48 ± 0.94^#^^	2.10 ± 0.97^*^	1.98 ± 1.08^*^	<0.001^d,e^
CSF protein (mean ± SD, g/L)	2.03 ± 2.08	2.79 ± 2.66^#^^	1.50 ± 1.25^*^	1.66 ± 1.92^*^	<0.001^d,e^
Positive CSF bacterial culture (%)	21.8 (183/838)	22.7 (76/335)	18.4 (78/424)^^^	36.7 (29/79)^#^	0.001^a^
Positive blood bacterial culture (%)	29.6 (248/838)	40.3 (135/335)^#^	21.7 (92/424)^*^	26.6 (21/79)	<0.001^a^
Brain MRI (*n =* 804)					
Leptomeningeal gadolinium enhancement (%)	64.6 (519/804)	58.7 (183/312)^#^	70.9 (293/413)^*^^	54.4 (43/79)^#^	<0.001^a^
Subdural effusion (%)	19.8 (159/804)	12.2 (38/312)^#^	27.6 (114/413)^*^^	8.9 (7/79)^#^	<0.001^a^
Parenchymal involvement (%)	11.9 (96/804)	16.0 (50/312)^#^	8.0 (33/413)^*^^	16.5 (13/79)^#^	0.002^a^
EEG (*n =* 618)					
Slow wave (%)	18.6 (115/618)	12.1 (26/214)^^^	15.8 (54/341)^^^	55.6 (35/63)^*#^	<0.001^a^
Epileptic discharge (%)	16.8 (104/618)	23.8 (51/214)^#^^	15.2 (52/341)^*^^	1.6 (1/63)^*#^	<0.001^a^
Severe and profound hearing impairment (%)	6.5 (36/557)	8.1 (13/160)^#^	4.0 (13/324)^*^^	13.7 (10/73)^#^	<0.001^a^
Complications					
Subdural effusion (%)	19.8 (159/804)	12.2 (38/312)^#^	27.6 (114/413)^*^^	8.9 (7/79)^#^	<0.001^a^
Ependymitis (%)	0.7 (6/803)	1.6 (5/312)	0.2 (1/413)	0.0 (0/79)	0.078^a^
Hyponatremia (%)	34.6 (290/838)	34.9 (117/335)	33.5 (142/424)	39.2 (31/79)	0.607^a^
Ventriculomegaly (%)	9.3 (75/804)	12.2 (38/312)	7.3 (30/413)	8.9 (7/79)	0.078^a^
GOS = 5 points (%)	92.0 (771/838)	90.4 (303/335)^#^^	95.8 (406/424)^*^^	78.5 (62/79)^*#^	<0.001^a^
Sequela (%)	10.7 (81/756)	10.5 (30/287)	10.1 (40/396)	15.1 (11/73)	0.444^a^

*, statistically significant difference compared with neonate patients; #, statistically significant difference compared with patients aged [29 days, 12 months); ^, statistically significant difference compared with patients aged ≥12 months; CSF, cerebrospinal fluid; CRP, C-reactive protein; EEG, electroencephalogram; GOS, Glasgow Outcome Scale; WBC, white blood cell count; IQR, interquartile range; SD, standard deviation; #, 70 patients with bacterial meningitis caused by *Streptococcus agalactiae*, *Streptococcus pneumoniae*, or *Escherichia coli* from 2015 to 2018 in the neurology department in one of our branch hospitals has been reported in a previous study (Chen et al., 2022). The 70 patients included neonate (*n =* 27) with *Streptococcus agalactiae* (*n =* 15) or *Escherichia coli* (*n =* 12), patients aged [29 days, 12 months) (*n =* 34) with *Streptococcus agalactiae* (*n =* 12), *Streptococcus pneumoniae* (*n =* 3) or *Escherichia coli* (*n =* 19) and patients aged ≥12 months (*n =* 9) who were all with *Streptococcus pneumoniae*.

a Chi-square test.

b Kruskal–Wallis H test.

c *Post hoc* by Nemenyi test.

d Analysis of variance.

e *Post hoc* by Student–Newman–Keuls *q* test.

#### 3.3.2 Initial CSF test results

Initial CSF WBC: For all patients, the median CSF WBC was 150 × 10^6^/L (IQR 40 × 10^6^/L, 650 × 10^6^/L) (normal reference range: 0–15 × 10^6^/L). Furthermore, 88.1% (738/838) of patients had CSF WBC higher than ULN, among which 52.6% (388/738) had neutrophils predominating, and CSF WBC belonged to (15 × 10^6^/L, 100 × 10^6^/L] in 30.1% (252/738), (100 × 10^6^/L, 500 × 10^6^/L] in 29.2% (245/738), (500 × 10^6^/L, 1,000 × 10^6^/L] in 8.8% (74/738), and >1,000 × 10^6^/L in 19.9% (167/738). The CSF WBC in neonate patients and patients aged ≥12 months was significantly higher than in patients aged [29 days, 3 months), and there was no significant difference of the CSF WBC between neonate patients and patients aged ≥12 months (more details are shown in **Table 1**).

A total of 100 patients (11.9%) showed normal CSF WBC in combination with CSF protein increase and/or glucose decrease, while 63.0% of them received antibiotic treatment before the first CSF test and 84% were younger than 3 months with common symptoms like fever, lethargy, irritability, anorexia, and poor feeding. In brain MRI, 70% (70/100) showed leptomeningeal gadolinium enhancement, 14% (14/100) showed subdural effusion, and 3% (3/100) showed brain parenchymal involvement. Among the patients, 29.0% (29/100) were positive in bacterial culture, including a positive result in blood culture (*n =* 22) for *Streptococcus agalactiae* (*n =* 9), *Klebsiella pneumoniae* (*n =* 5), and *Staphylococcus* (*n =* 5) that was commonly seen and a positive result in CSF culture (*n =* 11) for *Staphylococcus* (*n =* 4), *Streptococcus agalactiae* (*n =* 2), and *Enterococcus faecalis* (*n =* 2) that was commonly revealed. Moreover, 42% (42/100) of the patients showed CSF WBC higher than ULN in subsequent CSF examinations, with a median CSF WBC which was 34 × 10^6^/L (IQR 23 × 10^6^/L-50 × 10^6^/L), among which CSF WBC higher than ULN at 1 week after the initial CSF test was most commonly seen in 83.3% (35/42), followed by 2 and 3 weeks after the initial CSF test, seen in 9.5% (4/42) and 7.1% (3/42), respectively. The CSF WBC in 58 patients was always within normal range, among which 33 patients showed leptomeningeal gadolinium enhancement in brain MRI and 14 patients had positive bacterial culture, including 10 patients positive for blood culture with *Streptococcus agalactiae* as the most commonly seen, followed by *Staphylococcus* and four patients for CSF culture with *Staphylococcus* as the most commonly found.

Initial CSF glucose: For all patients, the initial CSF glucose was 1.76 ± 1.11 mmol/L (normal reference range: 2.8–4.2 mmol/L), and the CSF glucose was lower than LLN in 86.8% (727/838) and lower than 2.2 mmol/L in 57.8% (484/838). The CSF glucose in neonate patients was significantly lower than in patients aged [29 days, 12 months) and patients aged ≥12 months. There was no significant difference in the CSF glucose between patients aged [29 days, 12 months) and patients aged ≥12 months (more details are shown in **Table 1**).

The initial CSF glucose ≥2.8 mmol/L was seen in 111 patients (13.2%) accompanied by CSF protein increase and/or WBC increase, among which 15.3% (7/111) had the ratio of CSF glucose to peripheral blood glucose ≤0.4. Moreover, 78.4% (87/111) of these patients received antibiotic treatment before the first CSF test, and 85.6% (95/111) of these patients were younger than 1 year old. In brain MRI, 60.4% (67/111) showed leptomeningeal gadolinium enhancement, 16.2% (18/111) showed subdural effusion, and 5.4%% (6/111) showed brain parenchymal involvement. Among the patients, 29.7% (33/111) were positive in bacterial culture, including a positive result in blood culture (*n =* 24) for *Streptococcus agalactiae* (*n =* 7), *Escherichia coli* (*n =* 4), and *Streptococcus pneumoniae* (*n =* 3) as the most commonly seen and a positive result in CSF culture (*n =* 29) for *Streptococcus agalactiae* (*n =* 8) and *Streptococcus pneumoniae* (*n =* 6) as the most commonly revealed.

CSF glucose was lower than LLN in subsequent CSF examinations as seen in 88.3% (98/111) of patients and lower than 2.2 mmol/L in 28.8% (32/111), among which CSF glucose lower than LLN within 1 week after the initial CSF test was most commonly seen in 82.7% (81/98) and 1 week after the initial CSF test as seen in 17.3% (17/98). The CSF glucose in 13 patients was always within the normal range. Among these 13 patients, six patients (46.2%, 6/13) showed leptomeningeal gadolinium enhancement, two patients (15.4%, 2/13) showed subdural effusion in brain MRI, and four patients showed positive in bacterial culture, including *Escherichia coli* (*n =* 1) in bacterial blood culture and *Streptococcus pneumoniae* (*n =* 2) and *Haemophilus influenzae* (*n =* 1) in CSF bacterial culture.

Initial CSF protein: For all patients, the initial CSF protein was 2.03 ± 2.08 g/L (normal reference range: 0.15–0.45 g/L), and 96.9% (812/838) of patients had CSF protein higher than ULN, including CSF protein (0.45 g/L, ≤1.0 g/L] in 30.4% (255/838), (1.0 g/L, ≤2.0 g/L] in 41.3% (355/838), and >2.0 g/L in 27.3% (222/838). The CSF protein in neonate patients was significantly higher than in patients aged [29 days, 12 months) and patients ≥12 months (more details are shown in **Table 1**).

The initial CSF protein ≤0.45 g/L was seen in 26 patients (3.1%) accompanied by CSF WBC increase and/or CSF glucose decrease. Among these 26 patients, 80.8% (21/26) received antibiotic treatment before the first CSF test, 73.1% (19/26) were younger than 1 year old, and 26.9% (7/26) were positive for bacterial culture, including *Streptococcus pneumoniae* (*n =* 3), *Streptococcus agalactiae* (*n =* 2), *Escherichia coli* (*n =* 1), and *Haemophilus influenzae* (*n =* 1). At about 7 to 12 days after the first CSF test, 30.8% (8/26) of patients showed CSF protein higher than ULN, among which three patients were positive for bacterial culture including *Escherichia coli* (*n =* 2) and *Streptococcus agalactiae* (*n =* 1).

#### 3.3.3 Bacterial culture results

Among the patients, 38.7% (324/838) were positive in bacterial culture, including positive blood bacterial culture in 21.8% (183/838) and positive CSF bacterial culture in 29.6% (248/838). A total of 107 patients (32.9%, 107/324) were positive in both blood and CSF bacterial culture, among which 103 patients (96.3%, 103/107) had the same bacteria in blood culture and CSF culture. The blood culture and CSF culture were different for bacteria in four patients (0.5%, 4/107), of which three patients were neonates, and the results of the blood cultures in the other hospital before their admission to our hospital revealed *Streptococcus agalactiae*, *Escherichia coli*, and *Staphylococcus epidermidis* respectively, and the CSF culture results in our hospital revealed *Staphylococcus epidermidis* and *Staphylococcus epidermidis* and *Escherichia coli*, respectively. The remaining patient was a 4-month-old infant, and our hospital’s blood culture and CSF culture results revealed *Staphylococcus epidermidis* and *Salmonella agona*, respectively.

CSF bacterial culture: Among the patients, 21.8% (183/838) were positive in CSF bacterial culture, including positive results from the other hospital before admission to our hospital (*n =* 34) and from our hospital (*n =* 149). The pathogenic bacteria of the 183 children with positive CSF bacterial culture were mainly *Streptococcus agalactiae* (27.3%, 50/183), *Escherichia coli* (26.8%, 49/183), and *Streptococcus pneumoniae* (23.0%, 42/183) (more details are shown in **Supplementary Table S1**). Another 42 patients were positive for CSF bacterial culture with less common pathogenic bacteria (shown in **Supplementary Table S1**). The CSF bacterial culture positive rate in patients receiving antibiotic treatment before CSF examination was significantly lower than in patients who were not (14.1% vs. 25.1%, *P* < 0.0001). The CSF bacterial culture positive rate in patients aged ≥12 months was significantly higher than that in patients aged [29 days, 12 months) but was not different from that in neonates. Furthermore, the CSF bacterial culture positive rate in neonate patients was also not different from that in patients aged [29 days, 12 months) (more details are shown in **Table 1**).

**Figure 2:**
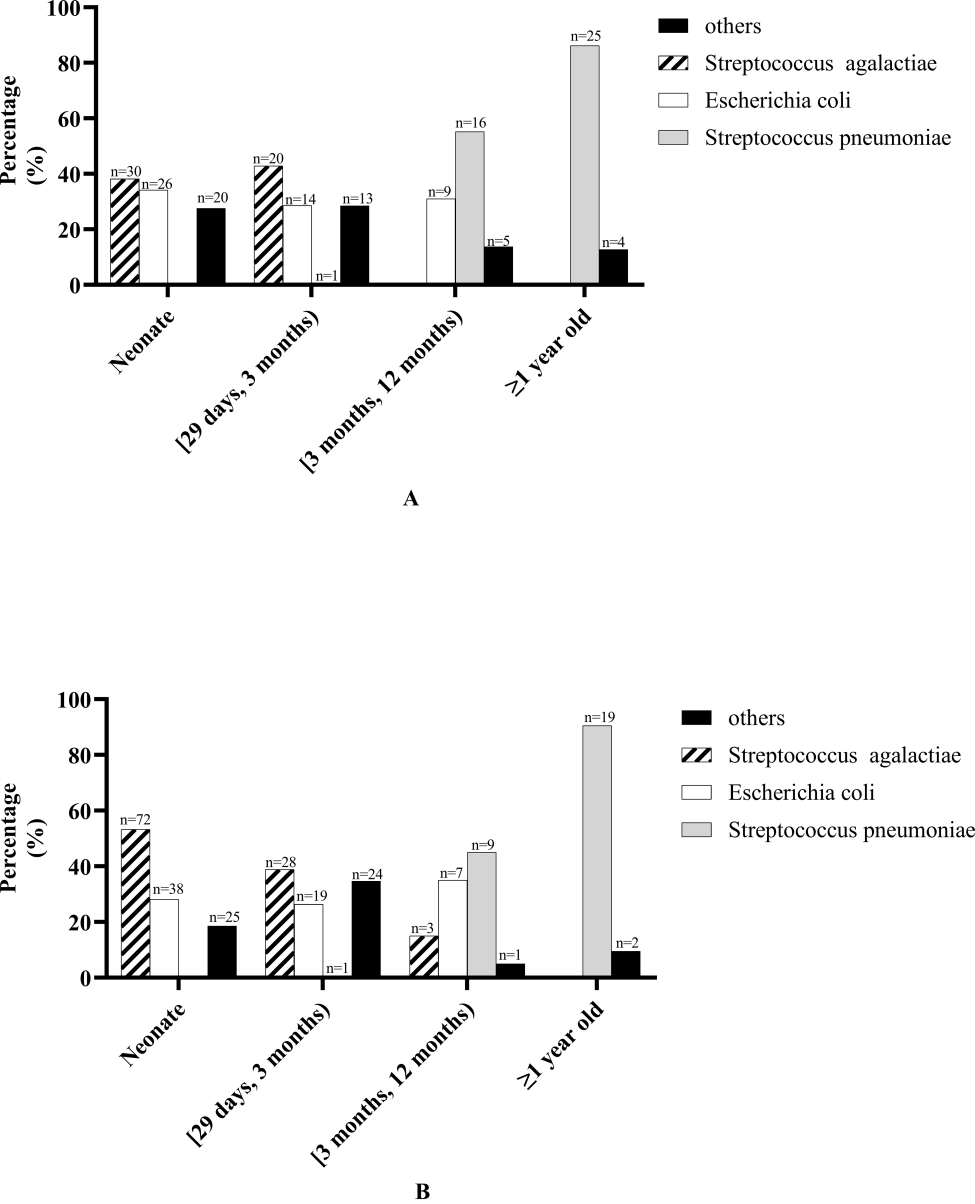
Bacterial culture results in patients of different ages. **(A)** Cerebrospinal fluid bacterial culture results in patients of different ages. “Other” bacteria in neonate including *Streptococcus pasteurianus* (*n =* 1), *Staphylococcus epidermidis* (*n =* 3), *Enterobacter aerogenes* (*n =* 1), *Acinetobacter baumannii* (*n =* 1), *Klebsiella pneumoniae* (*n =* 2), *Enterococcus faecalis* (*n =* 1), *Staphylococcus aureus* (*n =* 1), *Achromobacter xylosoxidans* (*n =* 1), *Elizabethkingia meningosepticum* (*n =* 4), *Staphylococcus haemolyticus* (*n =* 2), *Enterococcus faecium* (*n =* 1), *Pseudomonas aeruginosa* (*n =* 1), *Serratia marcescens* (*n =* 1). “Other” bacteria in patients aged [29 days, 3 months) including *Salmonella agona* (*n =* 1), *Staphylococcus epidermidis* (*n =* 1), *Klebsiella pneumoniae* (*n =* 1), *Streptococcus pneumoniae* (*n =* 1), *Enterococcus faecalis* (*n =* 1), *Streptococcus gallolyticus* (*n =* 1), *Staphylococcus aureus* (*n =* 2), *Elizabethkingia meningosepticum* (*n =* 1), coagulase-negative *Staphylococcus* (*n =* 1), *Staphylococcus haemolyticus* (*n =* 1), *Enterococcus faecium* (*n =* 2), *Staphylococcus cohnii* (*n =* 1). “Other” bacteria in patients aged [3 months, 12 months) including *Salmonella agona* (*n =* 1), *Haemophilus influenzae*(*n =* 1), *Staphylococcus haemolyticus* (*n =* 1), *Pseudomonas aeruginosa* (*n =* 1), *Salmonella paratyphi B* (*n =* 1). “Other” bacteria in patients ≥1 year old including *Listeria monocytogenes* (*n =* 1), *Haemophilus influenzae* (*n =* 1), *Streptococcus milleri* (*n =* 1), *Sphingomonas paucimobilis* (*n =* 1). **(B)** Blood bacterial culture results in patients of different ages. “Other” bacteria in neonate including *Staphylococcus epidermidis* (*n =* 3), *Enterobacter aerogenes* (*n =* 1), *Listeria monocytogenes* (*n =* 1), *Klebsiella pneumoniae* (*n =* 6), *Staphylococcus aureus* (*n =* 1), *Elizabethkingia meningosepticum* (*n =* 5), *Proteus mirabilis* (*n =* 1), *Staphylococcus hominis* (*n =* 1), *Staphylococcus haemolyticus* (*n =* 1), *Staphylococcus schleiferi* (*n =* 2), *Enterobacter cloacae* (*n =* 1), *Serratia marcescens* (*n =* 2). “Other” bacteria in patients aged [29 days, 3 months) including *Staphylococcus epidermidis* (*n =* 2), *Klebsiella pneumoniae* (*n =* 5), *Enterococcus faecalis* (*n =* 1), *Staphylococcus aureus* (*n =* 4), *Elizabethkingia meningosepticum* (*n =* 1), *coagulase-negative Staphylococcus* (*n =* 2), *Proteus mirabilis* (*n =* 1), *Staphylococcus hominis* (*n =* 2), *Staphylococcus haemolyticus* (*n =* 3), *Enterococcus faecium* (*n =* 1), *Staphylococcus Warneri* (*n =* 1), *Serratia marcescens* (*n =* 1). “Other” bacteria in patients aged [3 months, 12 months) including *Staphylococcus hominis* (*n =* 1). “Other” bacteria in patients ≥1 year old including *Haemophilus influenzae* (*n =* 1), *Staphylococcus hominis* (*n =* 1).

CSF bacterial culture results in patients of different ages: In neonates and patients aged [29 days, 3 months), the most common pathogenic bacterium was *Streptococcus agalactiae*, followed by *Escherichia coli*; in patients aged [3 months, 12 months), it was *Streptococcus pneumoniae* that was most commonly seen, followed by *Escherichia coli*; in patients ≥12 months, *Streptococcus pneumoniae* accounts for the vast majority (details are shown in **Figure 2A**).

Blood bacterial culture: Among the patients, 29.6% (248/838) were positive in blood bacterial culture, including positive results from the other hospital before admission to our hospital (*n =* 96) and from our hospital (*n =* 152). The pathogenic bacteria of the 248 children with positive blood bacterial culture were mainly *Streptococcus agalactiae* (41.5%, 103/248), *Escherichia coli* (25.8%, 64/248), *Streptococcus pneumoniae* (11.7%, 29/248), and *Staphylococcus* (10.5%, 24/248) (more details are shown in **Supplementary Table S1**). Another 57 patients were positive for blood bacterial culture with less common pathogenic bacteria (shown in **Supplementary Table S1**). The blood bacteria culture positive rate in patients receiving antibiotic treatment before blood examination was significantly lower than that in patients who were not (11.3% vs. 31.9%, *P* < 0.0001). The blood bacterial culture positive rate in neonate patients was significantly higher than in patients aged [29 days, 12 months) but was not different from that in patients ≥12 months. Furthermore, there was no significant difference in the blood bacterial culture positive rate in patients aged [29 days, 12 months) and patients ≥12 months (more details are shown in **Table 1**).

Blood bacterial culture results in patients of different ages: In neonates and patients aged [29 days, 3 months), the most common pathogenic bacterium was *Streptococcus agalactiae*, followed by *Escherichia coli*; in patients aged [3 months, 12 months), *Streptococcus pneumoniae* was most commonly seen, followed by *Escherichia coli*; in patients ≥1 year old, *Streptococcus pneumoniae* accounts for the vast majority (details are shown in **Figure 2B**).

### 3.4 Neuroimaging examination

Among the patients, 96.0% (804/838) underwent brain MRI examination, and 80.1% (644/804) of the patients had abnormal brain MRI, with leptomeningeal gadolinium enhancement most commonly seen in 64.6% (519/804), followed by subdural effusion in 19.8% (159/804), brain parenchymal involvement in 11.9% (96/804), and ventriculomegaly in 9.3% (75/804). The frequency of leptomeningeal gadolinium enhancement, subdural effusion, and brain parenchymal involvement in patients aged [29 days, 12 months) was higher than that in neonate patients and patients ≥12 months, but there were no differences between neonate patients and patients ≥12 months (more details are shown in **Table 1**). The frequency of sequela in patients with brain parenchymal involvement was significantly higher in patients without brain parenchymal involvement (28.1% vs. 10.0%, *P* < 0.001). Only eight patients underwent brain CT examination, and abnormal brain CT results included cerebral edema (*n =* 3), hydrocephalus (*n =* 3), leptomeningeal enhancement (*n =* 3), and brain parenchymal involvement (*n =* 2). A total of 34 patients did not undergo brain MRI examination, among which four patients could not undergo it for death, three patients had severe conditions and unstable vital signs, 13 patients had poor compliance and discharge, and in the remaining 14 neonate patients it was for unknown reasons.

### 3.5 Neuroelectrophysiological examination

Among the patients, 73.7% (618/838) underwent EEG examination, and the EEG results showed abnormalities in 38.3% (237/618) of patients, with slow wave (18.6%,115/618) and epileptic discharge (16.8%,104/618) being commonly seen. In addition, clinical seizures were detected in 4.0% (25/618) of patients, electrical seizures in 3.4% (21/618), and low voltage in 2.4% (15/618). The frequency of slow wave and epileptic discharge in patients aged ≥12 months was significantly higher than in neonate patients and patients aged [29 days, 12 months), but there were no differences between neonate patients and patients aged ≥12 months (more details are shown in **Table 1**).

Among the patients, 87.4% (732/838) underwent audiological assessment, including otoacoustic emissions in 175 patients (20.9%,175/838), of which they were all neonate, and the results were all normal and BAEP in 557 patients (66.7%, 557/838). Moreover, 52.2% (385/737) of patients had abnormal BAEP, with threshold increase most commonly seen in 99.5% (383/385) of patients, followed by latency prolonged in 10.1% (39/385), threshold increase accompanied by latency prolonged in 9.6% (37/385), and only latency prolonged in two patients. According to the hearing impairment classification, 52.0% (383/737) of the patients had hearing impairment, and mild and moderate hearing impairment were mainly found in 24.7% (182/737) and 22.4% (165/737) of the patients, respectively. Besides that, severe and profound hearing impairment was seen in 4.9% (36/737). The ratio of patients with severe and profound hearing impairment in neonates and patients aged ≥12 months was significantly higher than in patients aged [29 days, 12 months), but there was no difference in neonates and patients aged ≥12 months (more details are shown in **Table 1**). Latency prolonged was most commonly seen in III wave together with the V wave (46.2%,18/39), followed by only the I wave (23.1%,9/39) and only the III wave (17.9%,7/39).

### 3.6 Diagnosis

Among all 838 patients, 38.7% (324/838) of the patients were diagnosed with BM with proven bacteria, 14.9% (125/838) of the patients were diagnosed with BM with negative bacterial culture, 34.8% (290/838) of the patients were diagnosed as probable BM, and 11.8% (99/838) of the patients were diagnosed as suspected BM.

### 3.7 Comorbidity

Ten patients (1.2%, 10/838) had CSF fistula, including CSF rhinorrhea (*n =* 9) and CSF otorrhea (*n =* 1). Furthermore, 70.0% (7/10) of these 10 patients experienced recurrent BM (more details are shown in **Supplementary Data S1**).

Three patients had immunodeficiency disease, among which one patient was diagnosed with severe immunodeficiency disease (T and B lymphocyte deficiency) and died during hospitalization, one patient was diagnosed as probable with primary immunodeficiency disease and was followed for 6.5 years with one recurrence at the 4th year of follow-up, and another patient was diagnosed as X-linked agammaglobulinemia and was followed for 2.5 years without recurrence, which was reported in our previous study (more details are shown in **Supplementary Data S2**) (Zhang et al., 2019).

Upon combining physical examination and MRI, the results showed that one patient had a cranial structural abnormality of occipital meningocele (*n =* 1), and four patients had spinal structural abnormalities, including sacrococcygeal latent hairy sinus (*n =* 2) and meningomyomyelocele (*n =* 2). Of these five patients with cranial or spinal structural abnormalities, three patients were positive for bacterial culture, including two patients for *Escherichia coli* and one patient for *Pseudomonas aeruginosa* with multidrug resistance.

### 3.8 Complications

Subdural effusion was found in 19.8% (159/804) of patients, most of which (73.9%, 117/159) were improved with conservative medical therapy, while the others (26.4%, 42/159) needed surgical intervention. Ependymitis was found in 0.7% (6/804) of patients, among which five were neonates and one was 3 months old. Besides that, ventriculomegaly was found in 9.3% (75/804) of patients, among which 42.7% (32/75) received ventricular shunt treatment. On examination upon admission, hyponatremia was found in 34.6% (290/838) of the patients. The frequency of subdural effusion in patients aged [29 days, 12 months) was significantly higher than in neonate patients and patients aged ≥12 months, but there was no difference in neonate patients and patients ≥12 months. There was no difference in ependymitis, ventriculomegaly, and hyponatremia between patients of different ages (more details are shown in **Table 1**).

### 3.9 Treatment

#### 3.9.1 Antibiotic treatment

**Figure 3:**
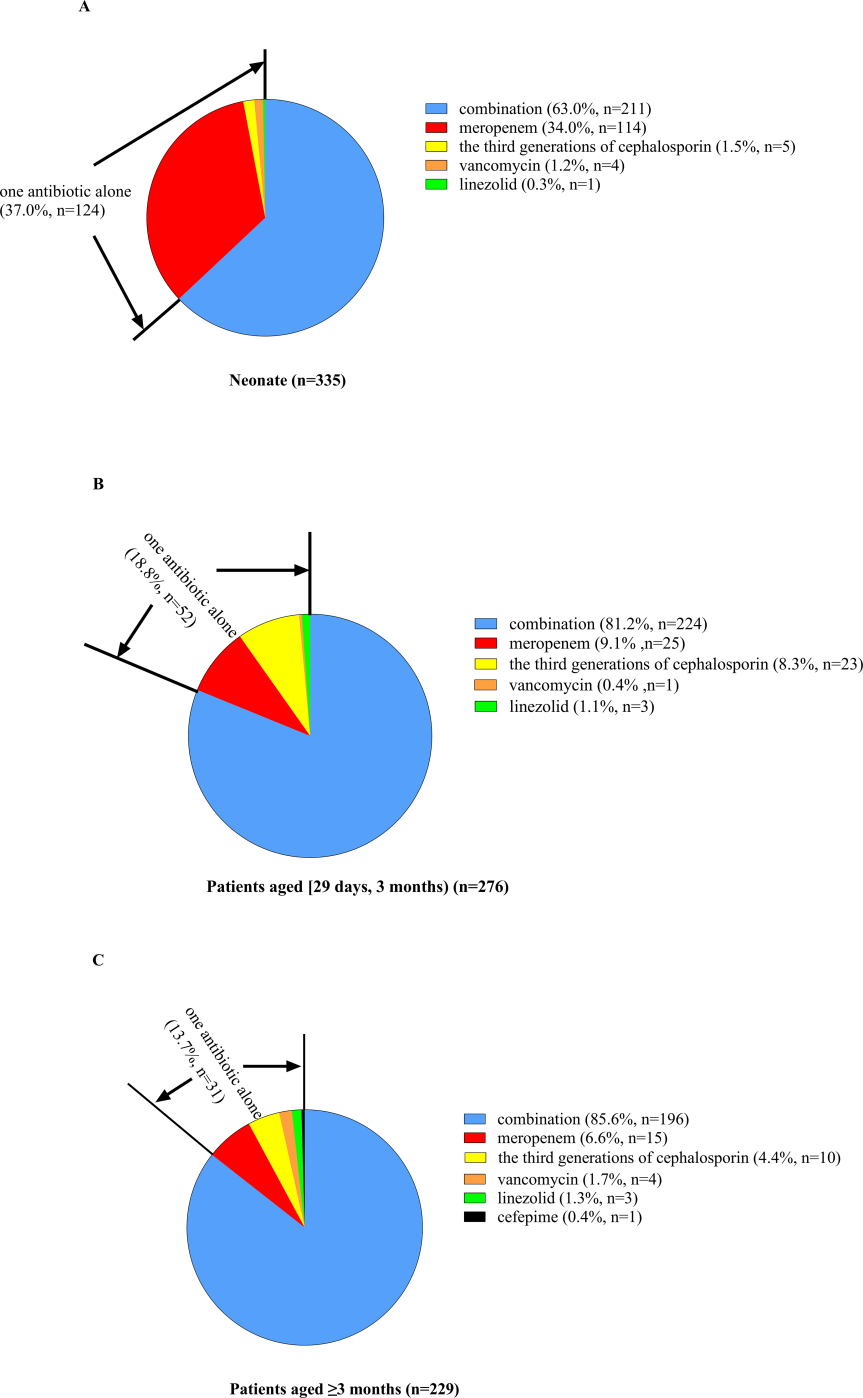
Percent of patients of different ages receiving antibiotic combination or alone treatment. **(A)** For neonate patients, 37.0% received one antibiotic alone, and the other 63.0% received antibiotic combination treatment. Among patients receiving only a single antibiotic, meropenem was the most commonly used, and the remaining antibiotics included the third generation of cephalosporin, vancomycin and linezolid. **(B)** For patients aged [29 days, 12 months], 18.8% received one antibiotic alone, and the other 76.4% received antibiotic combination treatment. Among patients receiving only a single antibiotic, meropenem was the most commonly used, followed by the third generation of cephalosporin, and the remaining antibiotics included vancomycin and linezolid. **(C)** For patients aged ≥3 months, 13.7% received one antibiotic alone, and the other 86.3% received antibiotic combination treatment. Among patients receiving only a single antibiotic, meropenem was the most commonly used, followed by the third generation of cephalosporin, and the remaining antibiotics included vancomycin (*n =* 4), linezolid, and cefepime.

**Figure 4:**
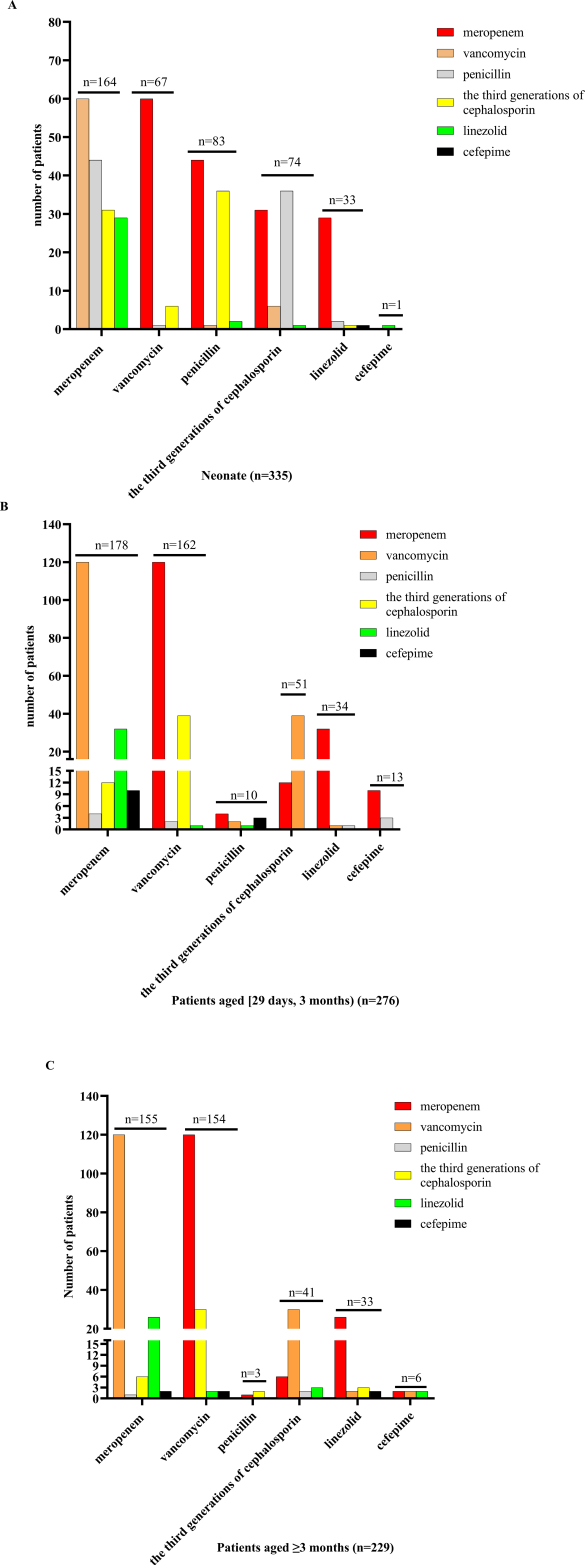
Antibiotic combination treatment in patients of different ages. **(A)** For neonate patients receiving antibiotic combination treatment, meropenem combination with another antibiotic was the most commonly used, followed by penicillin, and the remaining antibiotics included vancomycin, the third generation of cephalosporin, linezolid, and cefepime, and meropenem combination with vancomycin treatment was used mostly. **(B)** For patients aged [29 days, 12 months) who received antibiotic combination treatment, meropenem combination with another antibiotic was the most commonly used, followed by vancomycin, and the remaining antibiotics included penicillin, the third generation of cephalosporin, linezolid, and cefepime, and meropenem combination with vancomycin treatment was used mostly. **(C)** For patients aged ≥3 months receiving antibiotic combination treatment, meropenem combination with another antibiotic was the most commonly used, followed by vancomycin, and the remaining antibiotics included penicillin, the third generation of cephalosporin, linezolid, and cefepime, and meropenem combination with vancomycin treatment was used mostly.

Empiric antibiotic treatment was given to all patients before the causative bacteria were identified, and the antimicrobial agent was adjusted according to the susceptibility results if the pathogen was identified. The antibiotic treatment for patients of different ages is shown in **Figures 3** and **4**. For patients of different age groups, antibiotic combination treatment was more common than single antibiotic treatment, and meropenem in combination with another antibiotic was the most commonly used, especially meropenem in combination with vancomycin. Moreover, meropenem was also the most commonly used in single antibiotic treatment. Besides that, in non-neonate patients, the third-generation cephalosporin used alone is also common.

For all patients, the median duration of antibiotic treatment was 36 days (IQR: 25–36 days); for pathogen-bacteria-negative, it was 33 days (IQR: 23–54 days); for *Streptococcus agalactiae*, it was 58 days (IQR: 48.0–94.5 days), for *Escherichia coli*, it was 46 days (IQR: 34.75–64.5 days), and for *Streptococcus pneumoniae*, it was 36 days (IQR: 24.0–44.0 days).

#### 3.9.2 Other treatments

Dexamethasone (0.15 mg/kg day per 6 h for 4 days or 0.4 mg/kg day per 12 h for 2 days) was used in 12.3% (103/838) of the patients at the early acute stage. In addition, 9.5% (80/838) of the patients were admitted to ICU for advanced life support. Moreover, 9.9% (83/838) of the patients received surgical treatment, including subdural effusion drainage (*n =* 25), subdural effusion dissection (*n =* 17), excision of brain abscess (*n =* 3), ventriculoperitoneal shunting (*n =* 10), external ventricular drainage (*n =* 20), intracerebral hematoma evacuation (*n =* 6), myelomeningocele repair with release of tethered spinal cord (*n =* 2), and resection of pilose sinus in sacrococcygeal region (*n =* 1). For hearing impairment patients, two patients needed hearing aid installation treatment, among which one patient has already received hearing aid installation treatment, and six patients needed cochlear implantation, among which five patients received cochlear implantation and one was unable to have a cochlear implant installed due to severe cochlear polarization.

### 3.10 Outcome

For GOS at discharge, most of the patients (92.0%, 771/838) got 5 points, and between 2 and 4 points was seen in 1.2% (10/838) and 6.8% (57/838), respectively. The frequency of patients with GOS of 5 points was most commonly seen in patients aged [29 days, 12 months), which was 95.8%, followed by neonate patients with 90.4%, but in patients ≥12 months it was 78.5% (more details are shown in **Table 1**). Ten patients (1.2%, 10/838) died during hospitalization, among which six patients were positive for bacterial culture with *Streptococcus pneumoniae* (*n =* 2), and *Streptococcus agalactiae* (*n =* 2) was the most common bacterial pathogen, followed by *Escherichia coli* (*n =* 1) and *Staphylococcus aureus* (*n =* 1). The median time to death was 9 days (IQR: 5–16.5 days) after onset, and sepsis (*n =* 7) was the most common cause of death. The fatality rate during hospitalization in patients <1 year old was not different from patients >1 year old (1.2% vs. 1.3%, *P* = 0.611).

### 3.11 Follow-up

Of 828 patients who survived at discharge, 8% (66/828) of the patients were lost to follow-up. For 762 patients (92%) who were followed up, the median duration of follow-up was 52 months (IQR: 35–71 months, ranging from 10 to 95 months), and six patients gave up continuous treatment for poor compliance, and all of them died. Subsequently, the total fatality rate was 1.9% (16/838), and 10.7% (81/756) of the surviving patients at follow-up had sequela. The common sequela included delayed motor development (42.0%, 34/81), paralysis and/or dystonia (17.3%, 14/81), poor cognitive or intellectual disability (17.3%, 14/81), epilepsy (14.8%, 12/81), hearing impairment (14.8%, 12/81), with seven patients positive for *Streptococcus pneumoniae*, and speech disturbance (12.4%, 10/81). The less common sequela included epileptic attack only once (6.2%, 5/81), hyperactivity disorder (4.9%, 4/81), autism spectrum disorder (1.2%, 1/81), and visual disorder (1.2%, 1/81). There was no significant difference in the rate of sequela in patients of different ages (more details are shown in **Table 1**). Furthermore, 1.6% (12/756) of the surviving patients at follow-up experienced recurrent BM, with a total of 29 episodes of BM, including 19 BM episodes with proven bacteria, eight BM episodes with negative bacterial culture, and two BM episodes in a transferred patient without bacterial culture results of the first two episodes of BM in another hospital. Among 19 BM episodes with proven bacteria, *Streptococcus pneumoniae* was the most commonly seen in 68.4% (13/19), followed by *Escherichia coli* (*n =* 2) and *Staphylococcus epidermidis* (*n =* 1). The median onset age of patients experiencing recurrent BM was 6 years old (IQR: 2–7 years old). Moreover, 75.0% (8/12) of the patients experiencing recurrent BM had predisposing conditions, including seven patients with CSF fistula and one patient with X-linked agammaglobulinemia. Nine patients experienced one recurrence of BM, while the other three patients experienced two recurrences of BM, and all of them had CSF fistula. The median interval between the completion of therapy for the initial episode of BM to the second episode of BM was 1 month (IQR: 10–26 months, ranging from 0.75 to 36 months). Furthermore, 41.7% (5/12) of the patients experiencing recurrent BM had sequela.

### 3.12 Risk factors of ICU admission in the first hospitalization

A total of 80 patients (9.5%, 80/838) were admitted to ICU. On univariate analysis, the risk factors associated with admission to ICU were having been born prematurely, seizure, impaired consciousness, positive for *Streptococcus pneumoniae* in blood culture, blood WBC <4 × 10^9^/L, blood CRP >50 mg/L, initial CSF WBC >1,000 × 10^6^/L, initial CSF protein >2.0 g/L, and initial CSF glucose <1.0 mmol/L. On multivariate analysis, having been born prematurely, seizure, impaired consciousness, blood WBC <4 × 10^9^/L, blood CRP >50 mg/L, and initial CSF protein >2.0 g/L were independent risk factors of ICU admission (more details are shown in **Table 2**).

**Table 2:** Risk factors of ICU admission in the first hospitalization.

	Univariate analysis			Multivariate analysis		
	OR	OR (95%CI)	*P*-value	OR	95%CI	*P*-value
Born prematurely	2.388	1.427–3.996	0.001	2.606	1.399–4.853	0.003
Seizure	2.721	1.732–4.277	<0.001	2.161	1.272–3.672	0.004
Impaired consciousness	6.804	3.519–13.155	<0.001	5.838	2.599–13.116	<0.001
Blood bacterial culture						
*Streptococcus agalactiae*	1.836	0.969–3.479	0.062			
*Escherichia coli*	1.982	0.920–4.273	0.081			
*Streptococcus pneumoniae*	5.253	2.263–12.197	<0.001			
Blood WBC <4 × 10^9^/L	5.044	2.474–10.285	0.000	2.892	1.242–6.735	0.014
Blood CRP [50–100] mg/L	2.940	1.604–5.389	<0.001	2.238	1.119–4.475	0.023
Blood CRP >100 mg/L	2.607	1.562–4.351	<0.001	2.100	1.161–3.797	0.014
CSF						
WBC >1000×10^6^/L	3.455	2.110–5.650	<0.001			
Pro >2.0 g/L	6.114	3.219–11.612	<0.001	2.962	1.444-6.072	0.003
Glu <1.0 mmol/L	4.953	2.827–8.678	<0.001			

CI, confidence interval; CRP, C-reactive protein; CSF, cerebrospinal fluid; Glu, glucose; OR, odd ratio; Pro, protein; WBC, white blood cell count.

### 3.13 Risk factors of brain parenchymal involvement

Brain MRI found brain parenchymal involvement in 11.9% (96/804) of patients. On univariate analysis, the risk factors associated with brain parenchymal involvement were *Streptococcus pneumoniae* or *Streptococcus agalactiae* in blood bacterial culture, *Streptococcus pneumoniae*, *Escherichia coli*, or *Streptococcus agalactiae* in CSF bacterial culture, blood WBC <4 × 10^9^/L or ≥20 × 10^9^/L, blood CRP >50 mg/L, initial CSF WBC ≥500 × 10^6^/L, initial CSF protein >2.0 g/L, and initial CSF glucose <2.0 mmol/L. On multivariate analysis, blood WBC <4 × 10^9^/L, blood CRP >50 mg/L, initial CSF WBC >1,000 × 10^6^/L, and initial CSF protein >2.0 g/L were independent risk factors of brain parenchymal involvement (more details are shown in **Table 3**).

**Table 3:** Risk factors of brain parenchymal involvement.

	Univariate analysis			Multivariate analysis		
	OR	95%CI	*P*-value	OR	95%CI	*P*-value
Blood bacterial culture						
*Streptococcus agalactiae*	2.006	1.131–3.558	0.017			
*Streptococcus pneumoniae*	3.967	1.724–9.131	0.001			
CSF bacterial culture						
*Streptococcus agalactiae*	3.859	1.961–7.596	<0.001			
*Streptococcus pneumoniae*	3.893	1.888–8.027	<0.001			
*Escherichia coli*	2.462	1.140–5.317	<0.001			
Blood WBC <4 × 10^9^/L	6.777	3.398–13.514	<0.001	4.067	1.888–8.760	<0.001
Blood WBC ≥20 × 10^9^/L	2.082	1.149–3.774	0.016			
Blood CRP [50–100] mg/L	3.373	1.899–5.992	<0.001	2.159	1.147–4.063	0.017
Blood CRP >100 mg/L	3.032	1.855–4.954	<0.001	2.067	1.195–3.576	0.009
CSF						
WBC [500–1,000] × 10^6^/L	2.308	1.119–4.763	0.024			
WBC >1,000 × 10^6^/L	5.087	3.165–8.176	<0.001	2.002	1.123–3.571	0.019
Pro >2.0 g/L	9.333	4.851–17.958	<0.001	5.254	2.545–10.846	<0.001
Glu [1.0–2.0] mmol/L	2.239	1.271–3.945	0.005			
Glu <1.0 mmol/L	5.189	2.944–9.146	<0.001			

CI, confidence interval; CRP, C-reactive protein; CSF, cerebrospinal fluid; Glu, glucose; OR, odd ratio; Pro, protein; WBC, white blood cell count.

### 3.14 Risk factors of subdural effusion

Brain MRI found subdural effusion in 19.8% (159/804) of the patients. On univariate analysis, the risk factors associated with subdural effusion were as follows: patients aged [29 days, 1 year old), seizure, *Escherichia coli* in blood bacterial culture, *Escherichia coli* in CSF bacterial culture, and blood CRP >50 mg/L. On multivariate analysis, patients aged [29 days, 1 year old), seizure, *Escherichia coli* in blood bacterial culture, and blood CRP >50 mg/L were independent risk factors of subdural effusion (more details are shown in **Table 4**).

**Table 4:** Risk factors of subdural effusion.

	Univariate analysis			Multivariate analysis		
	OR	95%CI	*P*-value	OR	95%CI	*P*-value
Aged [29 days, 3 months)	2.779	1.819–4.248	<0.001	2.930	1.861–4.612	<0.001
Aged [29 days, 1 year old)	4.379	2.433–7.723	<0.001	4.400	2.355–8.223	<0.001
Seizure	1.973	1.384–2.811	<0.001	1.779	1.221–2.593	0.03
*Escherichia coli* in blood bacterial culture	2.532	1.458–4.397	0.001	3.018	1.630–5.586	<0.001
*Escherichia coli* in CSF bacterial culture	2.670	1.445–4.935	0.002			
Blood CRP (50–100] mg/L	1.847	1.032–3.303	0.039	1.862	1.007–3.441	0.047
Blood CRP>100 mg/L	2.559	1.604–4.083	<0.001	2.143	1.300–3.532	0.003

CI, confidence interval; CRP, C-reactive protein; OR, odd ratio.

### 3.15 Risk factors of severe and profound hearing impairment

Severe and profound hearing impairment was seen in 36 patients (6.5%, 36/557). On univariate analysis, the risk factors associated with severe and profound hearing impairment were as follows: patients aged ≥1 year old, impaired consciousness, movement disorder (paralysis and/or dystonia), vomiting, blood WBC >20 × 10^9^/L, initial CSF WBC >1000 × 10^6^/L, initial CSF glucose <1.0 mmol/L, initial CSF protein >2.0 g/L, *Streptococcus pneumoniae* or *Escherichia coli* in blood bacterial culture, and *Escherichia coli* in CSF bacterial culture. On multivariate analysis, movement disorder and *Streptococcus pneumoniae* or *Escherichia coli* in blood bacterial culture were independent risk factors of severe and profound hearing impairment (more details are shown in **Table 5**).

**Table 5:** Risk factors of severe and profound hearing impairment.

	Univariate analysis			Multivariate analysis		
	OR	95%CI	*P*-value	OR	95%CI	*P*-value
Aged ≥1 year old	4.251	1.784–10.132	<0.001	0.722	0.148–3.537	0.688
Impaired consciousness	6.271	2.249–17.486	<0.001	1.053	0.241–4.604	0.946
Movement disorder	15.893	5.552–45.496	<0.001	9.253	2.223–38.508	0.002
Vomiting	2.426	1.115–5.275	0.025	1.585	0.506–4.968	0.429
Blood WBC >20 × 10^9^/L	4.773	1.37–16.632	0.014	4.22	0.873–20.409	0.073
CSF WBC >1,000 × 10^6^/L	2.195	1.033–4.664	0.041	1.269	0.438–4.001	0.661
CSF Glu <1.0 mmol/L	2.794	2.501–6.246	0.012	1.112	0.309–4.001	0.871
CSF Pro > 2.0 g/L	2.709	1.098–6.683	0.031	2.034	0.51–8.112	0.314
*Escherichia coli* in blood bacterial culture	3.740	1.454–9.624	0.006	5.534	1.668–18.362	0.005
*Streptococcus pneumoniae* in blood bacterial culture	24.044	8.416–68.695	<0.001	7.465	1.282–43.486	0.025
*Escherichia coli* in CSF bacterial culture	14.077	5.503–36.010	<0.001	3.006	0.527–17.145	0.215

CI, confidence interval; CSF, cerebrospinal fluid; Glu, glucose; OR, odd ratio; Pro, protein; WBC, white blood cell count.

## 4 Discussion

We found that pediatric BM was more common in patients under 1 year old, accounting for 90.6%, especially newborns and infants under 3 months, accounting for 72.9%. The age of onset was similar to that of a previous study (Shinjoh et al., 2017). We also found that the high-occurrence season was summer (37.0%), which was similar to the study of Li et al. about adult and pediatric BM from four provinces in China, where the high-occurrence period was from July to August (Li et al., 2014). The high-occurrence period in our study was different from studies in other countries. BM peaks during the dry season in Africa (about from May to October in the south of the equator, October to May in the north of the equator), May to October in Brazil, and December to March in the USA, France, and the UK (Paireau et al., 2016). The differences in the high-occurrence periods across countries may be attributed to varying climate conditions. Studying the high-occurrence period is beneficial to develop prevention and control strategies for BM, but regional differences must be considered. CSF fistula and immunodeficiency are risk factors for BM (Brouwer et al., 2010; Zhang et al., 2019). In our study, 1.2% of the patients had CSF fistula, among which most (70.0%) had recurrent BM and 0.4% of the patients had immunodeficiency, in which both the recurrence and the fatality rate were as high as 33.3%.

In our study, fever was the most common symptom in patients of all ages. The second most common symptom in patients <3 years old was a seizure, while in patients ≥3 years old it was vomiting. However, in previous studies, fever was the most common symptom in BM patients, except for neonate patients, whose ratio of patients with fever was 6% to 39% (van de Beek et al., 2016b). In our study, fever was also the most common symptom in neonate patients, accounting for 86.0%. The high proportion of neonates with fever in our study may be associated with transferred patients in our study and our clinical practice in dealing with neonates with fever. In our study, 266 patients (27.0%, 226/838) were initially diagnosed with BM in other hospitals and then transferred to our hospital for poor treatment response, and some of them still had fever. For neonate patients with fever in our hospital, suggesting the CSF examination was determined by the risk of infection in the perinatal period, such as the duration of premature rupture of membranes before birth, testing for group B *Streptococcus*, and fever in pregnant mothers, as well as the presence of classic or atypical symptoms of BM, including dyspnea in neonate patients. Besides that, the frequency of vomiting increasing with age in our study was similar to that of another study (van de Beek et al., 2016b). A total of 70 patients with bacterial meningitis caused by *Streptococcus agalactiae*, *Streptococcus pneumoniae*, or *Escherichia coli* from 2015 to 2018 have been reported in a previous study which showed similar clinical features with the patients in this study (Chen et al., 2022).

The typical CSF change in BM are WBC and protein increase accompanied by glucose decrease. The CSF WBC often increases to 1,000–5,000 × 10^6^/L and can also be lower than <100 × 10^6^/L or higher than >10,000 × 10^6^/L, with neutrophils predominating and about 10% of the patients with lymphocyte predominating (Tunkel et al., 2004). In our study, the median CSF WBC was 150 × 10^6^/L (IQR: 40 × 10^6^/L,650 × 10^6^/L). Moreover, 88.1% of the patients had CSF WBC higher than ULN, among which 52.6% had neutrophils predominating. The majority of the patients in our study had typical CSF WBC increase, but only 52.6% of their WBC was neutrophils predominating. The early stage of BM or antibiotic treatment before CSF examination can cause lymphocyte predomination in CSF WBC classification (Visintin et al., 2010). In addition, 31.7% of the patients were transferred to our hospital and received antibiotic treatment before CSF examination, which may cause the related lower ratio of patients in our study to show neutrophils predominating in CSF WBC. Besides that, we found that 11.9% of the patients showed normal CSF WBC accompanied by CSF protein increase and/or CSF glucose decrease. Moreover, 42.0% of these patients showed a CSF WBC increase, among which 1 week after the initial CSF test was the most commonly seen in 83.3%. In previous studies, a normal or slight increase of CSF WBC was also found in BM patients with positive CSF bacterial culture, especially in patients with septic shock, which may be associated with the initial CSF sample taken before inflammation in brain initiation or the immune response suppressed under severe infection (Georget-Bouquinet et al., 2008; Heckenberg et al., 2008; van de Beek et al., 2016a). In our study, majority of the patients (84%) with normal initial CSF WBC were younger than 3 months, and their immune systems were immature. Additionally, antibiotic treatment prior to CSF examination may prevent a significant increase in CSF WBC in BM patients. It is worth noting that 63.0% of these 100 patients received antibiotic treatment. Therefore, BM should not be excluded based solely on normal CSF WBC for patients suspected of BM, especially for patients younger than 3 months. Comprehensive judgment should be made by combining factors of antibiotic treatment before lumbar puncture, other CSF examination results like protein and glucose, bacterial culture results, and brain MRI. Re-examination of CSF 1 week after the initial examination sometimes is often necessary.

The CSF glucose decrease in BM is related to both the bacterial consumption and inflammatory cells in the CSF as well as the reduced glucose transport capacity of the blood–brain barrier on the other (Viola, 2010; Baud et al., 2018). In our patients, the initial CSF glucose was 1.76 ± 1.11 mmol/L. The CSF glucose in neonate patients was significantly lower than in patients aged [29 days, 12 months) and patients aged ≥12 months. Moreover, the CSF WBC and protein in neonate patients were also significantly higher than in patients aged [29 days, 12 months). The CSF protein in neonate patients was also significantly higher than in patients aged ≥12 months. These findings suggest that the inflammatory response in neonate patients is more severe than in non-neonate patients, leading to lower CSF glucose in neonate patients. CSF glucose not lower than LLN was found in 13.2% of the patients, and CSF glucose lower than LLN in subsequent CSF examinations was seen in 88.3% of the patients, among which CSF glucose lower than LLN within 1 week after the initial CSF test was most commonly seen. Previous studies found normal CSF glucose in BM patients with positive CSF bacterial cultures (Garges et al., 2006; Georget-Bouquinet et al., 2008; Heckenberg et al., 2008). The initial CSF glucose within the normal range may be associated with the ability of the body to maintain glucose homeostasis in the early stage of the disease. In addition, we found that the initial CSF protein was 2.03 ± 2.08 g/L, and CSF protein increase was seen in most patients (96.9%), which was consistent with the typical CSF protein change in BM. Atypical CSF change can complicate the diagnosis of BM, and when interpreting the CSF examination, the patient’s age and antibiotic treatment should be considered. Typical CSF change may also be observed 1 week after the initial CSF examination for some patients.

Bacterial cultures in blood and CSF are important evidence for diagnosing BM. The positive rate of bacterial culture in our study was 38.7%, similar to the study of Guo et al., a retrospective study of pediatric BM from Beijing in China, which was 43.3% (Guo et al., 2016). Dalton et al. and Allison et al. found that antibiotic treatment before bacterial culture can reduce a positive bacterial culture significantly (Dalton and Allison, 1968). We also found that the CSF bacterial culture positive rate in patients receiving antibiotic treatment before CSF examination was significantly lower than in patients not receiving antibiotic treatment as well as in blood bacterial culture. Besides that, we found that the CSF bacterial culture positive rate in patients aged ≥12 months was significantly higher than that in patients aged [29 days, 12 months). The CSF WBC was positively related to the bacterial load (Puccioni-Sohler and Brandão, 2007). We found that the initial CSF WBC of the patients aged ≥12 months was significantly higher than in patients aged [29 days, 12 months). These suggested a higher positive CSF bacterial culture in patients ≥1 year old than in patients aged [29 days, 12 months), which may associate with a higher bacteria load in patients ≥1 year old.

The pathogen bacteria of pediatric BM varied by region, patient’s age, and immune status (van de Beek et al., 2016b). It was also affected by the meningitis vaccines used after the end of the 20th century (Parikh et al., 2020). We found that the pathogenic bacteria in CSF and blood were mainly *Streptococcus agalactiae*, *Escherichia coli*, and *Streptococcus pneumoniae*. The common pathogenic bacteria in our study were similar to that of a multicenter retrospective study of pediatric bacterial meningitis in Japan, in which the main pathogenic bacteria were also *Streptococcus agalactiae*, *Streptococcus pneumoniae*, and *Escherichia coli* (Shinjoh et al., 2020). For patients of different ages, we found that the common pathogenic bacteria in CSF and blood bacterial culture was the same: in neonates and patients aged [29 days, 3 months), the most common pathogenic bacteria were *Streptococcus agalactiae*, followed by *Escherichia coli*; in patients aged [3 months, 12 months), *Streptococcus pneumoniae* was most commonly seen, followed by *Escherichia coli*; in patients ≥1 year old, *Streptococcus pneumoniae* accounts for the vast majority. The main pathogenic bacteria in patients of different ages in our study was consistent with that of a multicenter retrospective study of pediatric bacterial meningitis in China (Li et al., 2018). In Europe, the USA, Canada, and other developed countries, the common pathogenic bacteria in newborns are *Streptococcus agalactiae*, *Escherichia coli*, and *Listeria monocytogenes* and in patients ≥1 month are *Streptococcus pneumoniae*, *Neisseria meningitidis*, and *Haemophilus influenzae* (Thigpen et al., 2011; Le Saux, 2014; van de Beek et al., 2016b). In contrast, we found only two patients positive for *Listeria monocytogenes* and three for *Haemophilus influenzae* and none for *Neisseria meningitis* (**Supplementary Table S1**). *Listeria monocytogenes*, *Haemophilus influenzae*, and *Neisseria meningitis* were also rare in BM patients in another study from China (Li et al., 2018). The different results of common pathogenic bacteria in patients of different ages that varied in our study against those in Europe, the USA, Canada, and other developed countries may be due to regional differences.

BM is an acute and devastating infection that can lead to severe sequela and mortality if diagnosis and treatment are delayed (Zainel et al., 2021). Therefore, timely and appropriate empiric antibiotic treatment before the causative bacteria were identified is important. The choice of empiric antibiotic treatment varied depending on region, antimicrobial resistance, and age of the patient (Sakata et al., 2010; Le Saux, 2014; Subspecialty Group of Neurology tSoPCMA, 2019). The BM treatment guidelines in China recommend that for neonate patients, ampicillin plus the third generation cephalosporin is used, and when drug-resistant *Escherichia coli* are considered, vancomycin plus meropenem is used (Subspecialty Group of Neurology tSoPCMA, 2019). On the other hand, for patients older than 1 month, the third generation of cephalosporin plus vancomycin is used, and when drug-resistant *Escherichia coli* is considered, vancomycin plus meropenem is used. In China, the resistance rate of *Escherichia coli* to the third generation of cephalosporin in tertiary hospitals is high, and the resistance rate of *Streptococcus pneumoniae*, which causes meningitis, to penicillin and the third generation of cephalosporin is also high (Li et al., 2018; Subspecialty Group of Neurology tSoPCMA, 2019). Our hospital was a tertiary hospital, and some of our patients received antibiotic treatment with the third generation of cephalosporin, most commonly before being admitted to our hospital and have not improved and then were transferred to our hospital (Li et al., 2018; Subspecialty Group of Neurology tSoPCMA, 2019). In our study, antibiotic combination treatment was more common than single antibiotic treatment, and meropenem combined with another antibiotic was the most commonly used, especially for meropenem combined with vancomycin for patients of different ages. Meropenem, frequently used in our study, was associated with the high resistance rate of bacteria in tertiary hospitals in China and proper treatment response for transferred patients.

Advanced life support is essential for BM patients for complications with septic shock, status epilepticus, or respiratory failure. Liang et al. found that the risk factor of admission to ICU included *Streptococcus pneumoniae*, leukopenia, and the ratio of CSF glucose to the peripheral blood glucose lower than 0.25 in BM patients with proven bacteria (Wee et al., 2016). We found on multivariate analysis that having been born prematurely, seizure, impaired consciousness, blood WBC <4 × 10^9^/L, blood CRP >50 mg/L, and initial CSF protein >2.0 g/L were independent risk factors of admission to ICU. Leukopenia as the risk factor of admission to the ICU was similar to the study of Liang et al., while the other risk factors of admission to the ICU in our study were different, which may be caused by the difference in study subjects. In other studies, having been born prematurely, seizure, and impaired consciousness were found to be the risk factors of death, suggesting that patients who were born prematurely, seizure, and impaired consciousness are more likely to present unstable vital signs and need advanced life support in ICU (Olson et al., 2015; Okike et al., 2018). Blood CRP and CSF protein are associated with the severity of inflammation reactions. For patients, especially newborns, severe inflammatory response to infection can lead to septic shock and increases the chance of ICU admission (Keane et al., 2015; Mohamed et al., 2017; Mao et al., 2018).

Brain parenchymal involvement in BM is associated with disease severity and sequela (Packer et al., 1982; Sellner and Leib, 2006; Oliveira et al., 2014). In previous studies of BM, brain parenchymal involvement was seen in 10% to 40% of the patients (Packer et al., 1982; Oliveira et al., 2014). The brain parenchymal involvement in our patients was 11.9%, and the frequency of sequela in patients with brain parenchymal involvement was significantly higher than in patients without. Besides that, we found that blood WBC <4 × 10^9^/L, blood CRP >50 mg/L, initial CSF WBC >1,000 × 10^6^/L, and initial CSF protein >2.0 g/L were independent risk factors of brain parenchymal involvement. These risk factors were associated with the severity of inflammation after infection and suggested that the inflammation extends to the brain’s parenchyma and induces cerebritis (Packer et al., 1982).

Subdural effusion is one of the most common complications of BM, and its frequency can be as high as 40%–50%, among which 20% need a surgical intervention (Oliveira et al., 2014; Hsu et al., 2018). In our study, 19.0% of the patients are complicated with subdural effusion, and 26.4% need a surgical intervention. Vasiliki et al. found that seizure, leukopenia, low CSF glucose, high CSF protein, and positive blood culture were risk factors of subdural effusion (Vasilopoulou et al., 2011). Our study found that patients aged [29 days, 1 year old), seizure, *Escherichia coli* in blood bacterial culture, and blood CRP >50 mg/L were independent risk factors of subdural effusion. The factors leading to subdural effusion development in BM are unknown. Various mechanisms, including arachnoid rupture, impaired blood–brain barrier function, and cerebral atrophy, may be involved in the development of subdural effusion (Lee et al., 1994). High CRP and *Escherichia coli* in blood bacterial culture were risk factors for subdural effusion that may be associated with blood–brain barrier function impairment caused by interleukin 6 or nitric oxide (Al-Obaidi and Desa, 2018; Liu et al., 2020).

Hearing impairment is another common sequela of BM, accounting for 5%–54% of the patients, among which 2%–5% were severe and with profound hearing impairment (Rodenburg-Vlot et al., 2016; van de Beek et al., 2016b; van Ettekoven et al., 2017). In our study, 52.2% of the patients had hearing impairment, and 4.9% had severe and profound hearing impairment. Hearing impairment in BM is associated with the types of infectious bacteria and is most commonly seen in patients with *Streptococcus pneumoniae* infection, in which it can be as high as 36.5% (Kutz et al., 2006). In addition, in previous studies, CSF glucose lower than 0.6 mmol/L, high CSF protein, and the presence of neurological deficits are risk factors for hearing impairment (Gupta, 1993). Among the risk factors of severe and profound hearing impairment, besides movement disorder and *Streptococcus pneumoniae* being reported in previous studies, we found that *Escherichia coli* in blood bacterial culture was also a risk factor.

In our study, hyponatremia was found in 34.6% of the patients on admission examination in our hospital, which was lower than that in the study of Zheng et al. about hyponatremia in children with BM, which was 66.4% (Zheng et al., 2019). Furthermore, 31.7% (266/838) of the patients in our study were initially diagnosed with BM in other hospitals and then transferred to our hospital. That the hyponatremia in some of these transferred patients has been corrected before their admission to our hospital may account for the lower ratio of patients with hyponatremia in our study. In total, hyponatremia is commonly seen in children with BM. Besides that, the study of Zheng et al. found that hyponatremia was associated with the severity of pediatric BM. Hyponatremia should be checked in pediatric BM (Zheng et al., 2019).

Recurrent BM is rarely seen, accounting for 1.2%–6% of meningitis episodes (Tebruegge and Curtis, 2008). In our study, 1.6% of surviving patients at follow-up experienced recurrent BM. Most patients with recurrent BM have predisposing recurrent conditions, including congenital or acquired anatomical defects, congenital or acquired immunodeficiency, and chronic para-meningeal infections (Tebruegge and Curtis, 2008). Anatomical defects were the most common predisposing conditions for recurrent BM, accounting for nearly two-thirds (Tebruegge and Curtis, 2008; Chen et al., 2021). In our study, 75% of the patients experiencing recurrent BM had predisposing recurrent conditions, with CSF fistula being the most commonly seen. *Streptococcus pneumoniae* was the most common pathogenic bacteria in episodes of recurrent BM, accounting for half to more than 80% (Tebruegge and Curtis, 2008; Chen et al., 2021). Similarly, *Streptococcus pneumoniae* was also the most common pathogenic bacteria in episodes of recurrent BM.

In our study, 92.0% of the patients had a GOS at 5 points, indicating that most recovered well at discharge. In our study, 16 patients (1.9%, 16/838) died, including 10 patients who died at hospitalization and six patients who gave up treatment for poor compliance and died after discharge. The World Health Organization reported that the fatality rate of BM with adult and pediatric patient involvement was about one-sixth (WHO, 2023). The fatality rate of pediatric BM is 0.8%–3%, and the fatality rate of adult BM is more than 10 times that of children (Køster-Rasmussen et al., 2008; Guo et al., 2016). The fatality rate of BM is affected by the regional economic level, the type of pathogenic bacteria, study subjects’ involvement, and the time to initiation of treatment (Brouwer et al., 2010). The relatively lower fatality rate in our study was related to the subjects being children and the timely use of antibiotics. The guidelines in several countries emphasize the need for early antibiotic therapy in patients with BM (Tunkel et al., 2004; Chaudhuri et al., 2008; Køster-Rasmussen et al., 2008; Le Saux, 2014; van de Beek et al., 2016b; Posadas and Fisher, 2018). In clinical practice, awareness of the diagnosis of BM and timely initiation of empiric antibiotics are gradually increasing among clinicians.

This study has several limitations. Firstly, it is a single-center, retrospective study, which may introduce selection bias and limit the generalizability of the findings. Secondly, the sample size, though relatively large, may still lead to an underestimation or overestimation of certain risk factors. Additionally, due to the retrospective nature of the data collection, causal relationships cannot be definitively established. These limitations should be considered when interpreting the results, and future prospective, multi-center studies would help to address these concerns.

## 5 Conclusions

Most pediatric BM patients in Southern China are under 1 year of age, with more distribution among male patients and age-related differences in clinical features and outcomes. Recurrent BM is rare but more likely in patients with underlying conditions such as CSF fistula or immunodeficiency. Most patients have favorable outcomes, with a low fatality rate; however, approximately 10% of the survivors experience neurological sequelae. Several clinical risk factors were identified, including those associated with ICU admission, brain parenchymal involvement, and hearing impairment. These findings provide valuable insights into the clinical management and risk stratification of pediatric BM in this region.

## Data Availability

The original contributions presented in the study are included in the article/**Supplementary Material**. Further inquiries can be directed to the corresponding author.
