# Supplementary material for: Pediatric bacterial meningitis in southern China: analysis of 838 cases
Source: Front Cell Infect Microbiol. 2025 Feb 5;15:1481716. doi: 10.3389/fcimb.2025.1481716 (PMC11835870; doi:10.3389/fcimb.2025.1481716)
Supplement: Supplementary file 1 [file DataSheet1.docx]

Supplementary Material

# Supplementary Data

## Supplementary Data 1

**Ten patients with CSF leakage**

Ten patients (1.2%,10/838) were combined with CSF leakage, including CSF rhinorrhea (n=9) and CSF otorrhea (n=1). 3 patients had CSF leakage before bacterial meningitis onset, among which 2 patients were combined with Mondini dysplasia and received CSF leakage repair. 3 patients were found to have CSF leakage at the onset of bacterial meningitis, and the routine and biochemical results of the leaked fluid were similar to those of CSF during the same period, and two patients underwent CSF leakage repair. 4 patients were found to have CSF leakage during the recurrent bacterial meningitis, among which 3 patients received CSF leakage repair and one patient lost follow-up for poor compliance. 70.0% (7/10) of these ten patients experienced recurrent bacterial meningitis. In total, 7 patients received CSF leakage repair with a median postoperative follow-up of 45 months (IQR 36-61 months), and two patients relapsed (28.6%, 2/7). Two patients have not received CSF leakage repair for no structural defects found by imaging examinations, among which one patient relapsed at 5 years of follow-up, and one patient did not relapse follow up for 17 months.

## Supplementary Data 2

**Three patients diagnosed with immunodeficiency disease**

One patient showed an obvious decrease of T lymphocyte, nature killer (NK) cells and B lymphocyte in peripheral blood lymphocyte count. And whole exome sequencing (WES) results showed adenylate deaminase gene mutation (Chr20 exon 10 c.905C>T & exon 8 c.747dupT). After an immunology consultation, this patient was diagnosed with severe combined immunodeficiency disease (T and B lymphocyte deficiency).

One male patient had a recurrent respiratory infection, tympanitis and sinusitis before onset, and his serum immunoglobin G (IgG), IgA, and immunoglobin M (IgM) decreased at admission and two follow-ups one year after discharge. However, his peripheral lymphocyte count included total lymphocyte, T lymphocyte, B lymphocyte and NK cells were within the normal range. And WES results showed no abnormalities. He was diagnosed as probable with primary immunodeficiency disease after an immunology consultation.

Another male patient showed an obvious decrease in IgG and IgM, but his IgA was within normal range. His peripheral lymphocyte count showed that T lymphocyte was within normal range, but B lymphocyte and NK cell decreased. WES result showed Bruton’s tyrosine kinase gene mutation (Chr X c.1349+5G>A). He was diagnosed with X-linked agammaglobulinemia after an immunology consultation.

# Supplementary Figures and Tables

## Supplementary Table 1

**bacterial culture results**

| Positive for CSF bacterial culture (n=183) | | | |
| --- | --- | --- | --- |
| *Streptococcus agalactiae* | n=50 | *Pseudomonas aeruginosa* | n=2 |
| *Escherichia coli* | n=49 | *Haemophilus influenzae* | n=2 |
| *Streptococcus pneumoniae* | n=42 | *Enterococcus faecalis* | n=2 |
| *Staphylococcus haemolyticus* | n=4 | *Salmonella agna* | n=2 |
| *Staphylococcus epidermidis* | n=4 | *Salmonella paratyphi type B* | n=1 |
| *Staphylococcus aureus* | n=3 | *Listeria monocytogenes* | n=1 |
| *Staphylococcus cohnii* | n=1 | *Enterobacter aerogenes* | n=1 |
| *coagulase-negative Staphylococcus* | n=1 | *Acinetobacter baumannii* | n=1 |
| *Elizabethkingia meningosepticum* | n=5 | *Serratia marcescens* | n=1 |
| *Klebsiella pneumoniae* | n=3 | *Streptococcus pasteurianus* | n=1 |
| *Enterococcus faecium (group D)* | n=3 | *Streptococcus gallolyticus* | n=1 |
| *Achromobacter xylosoxidans* | n=1 | *Streptococcus milleri* | n=1 |
| *Sphingomonas paucimobilis* | n=1 |  |  |
| Positive for blood bacterial culture (n=248) | | |  |
| *Streptococcus agalactiae* | n=103 | *Elizabethkingia meningosepticum* | n=6 |
| *Escherichia coli* | n=64 | *Klebsiella pneumoniae* | n=11 |
| *Streptococcus pneumoniae* | n=29 | *Serratia marcescens,* | n=3 |
| *Enterobacter aerogens* | n=1 | *Proteus mirabilis* | n=2 |
| *Listeria monocytogenes* | n=1 | *Enterobacter cloacae* | n=1 |
| *Haemophilus influenzae* | n=1 | *Enterococcus Faecium* | n=1 |
| *Enterococcus faecalis* | n=1 |  |  |
| *Staphylococcus* | n=24: *Staphylococcus aureus* (n=5), *Staphylococcus* [*hominis*](http://www.baidu.com/link?url=TOeZ-k23R72sd986xuOfDwW8rYByRA-a4wLYIkwUi2yIXZfpHjLyeiqMfpnAVwXHg0oFqSpu9tpVFGHV7RMz5_KvXt5xYxGfb9iRCrHdaquovFwDM21V9HZJIn488wE3) (n=5), *Staphylococcus epidermidis* (n=5), *Staphylococcus haemolyticus* (n=4), *Staphylococcus schleiferi* (n=2), coagulase-negative *Staphylococcus* (n=2), and *Staphylococcus Worth* (n=1) | | |
